# Draft genome sequences of five *Stenotrophomonas indicatrix* strains isolated from soil

**DOI:** 10.1128/mra.00656-23

**Published:** 2024-04-02

**Authors:** Joanna Żur-Pińska, Vrinda Sharma, Anthony G. Hay

**Affiliations:** 1 Department of Microbiology, Cornell University, Ithaca, New York, USA; University of Strathclyde, Glasgow, United Kingdom

**Keywords:** *Stenotrophomonas*, soil microbiology, triclosan, antibiotic resistance, biodegradation

## Abstract

Here, we present genome sequences of five *Stenotrophomonas indicatrix* strains, isolated from agricultural soil. *Stenotrophomonas* strains are commonly associated with the rhizosphere and are well-known for their ability to degrade xenobiotics. Yet, to date, knowledge about *S. indicatrix* is limited.

## ANNOUNCEMENT

*Stenotrophomonas* is a diverse genus of Gram-negative γ-proteobacteria that includes opportunistic pathogens (1, 2). *S. indicatrix* strains have been reported to degrade phenanthrene (3). An increase in the abundance of *Stenotrophomonas* 16S rRNA genes has also been reported in soils containing triclosan (TCS) (4). TCS is a biocide found in wastewater, biosolids, and soils receiving biosolids (5), reported to select multi-drug resistant bacteria in the environment. In *Stenotrophomonas maltophilia*, TCS induces the expression of SmeDEF, an efflux pump responsible for antibiotic resistance (6).

All *Stenotrophomonas* strains described here were isolated from the plow layer (0–15 cm) of an agricultural soil (silty clay loam) collected from Dilmun Hill Student Farm (Cornell University, Ithaca, NY, USA; 42.44890697063454, –76.45800728579064) in February 2020. Three 200 g samples were composited, then 3 × 50 g subsamples were incubated in 250 mL Mason jars after being brought to 50% of their water-holding capacity. After 3 weeks of incubation in the dark at 22°C, the soil samples were serially diluted in phosphate-buffered saline and spotted on vancomycin-imipenim-amphoterocin (VIA) plates, described as selective for *S. maltophilia* (7). The plates were then incubated in the dark at room temperature for 5 days. Well-isolated colonies from the lowest dilution supporting growth were restreaked on lysogeny broth (LB) three times to obtain pure colonies. All colonies also grew on LB containing 10 mg L^−1^ TCS.

For sequencing, individual colonies of five different original isolates were inoculated into 3 mL of LB and incubated at 37°C for 18 h, centrifuged, and then subjected to DNA extraction (ZymoDNA, Macherey-Nagel, USA). Separate DNA libraries were prepared for each strain using the Nextera XT DNA Library Preparation Kit (Illumina Inc., San Diego, CA, USA) according to the manufacturer’s protocol and sequenced using the Illumina NextSeq platform with 2 × 150 paired-end reads at the Cornell University sequencing facility.

Default software parameters were used for processing sequencing reads, assembling and annotating genomes, and determining their taxonomy. The raw paired-end Fastq reads were quality checked using FastQC v.0.11.7 (8) and low-quality bases were trimmed using Trim Galore! (v.0.6.7) (9). The trimmed reads were assembled using SPAdes v.3.15.4 (10). The assembled draft genomes were annotated using the NCBI Prokaryotic Genome Annotation Pipeline v.6.5 (11). The quality of assemblies and annotations was checked by BUSCO Assessing Genome Assembly and Annotation Completeness (v.5.4.6) and Quast Genome Assembly Quality (v.5.2.0) (12, 13). Detailed taxonomic classification, performed with FastANI v.1.33 queried the *S. indicatrix* WS40 (GCF_002750975.1) sequence and classified all strains as *S. indicatrix* (14). The summary report is given in Table 1. Based on BUSCOs, the genomes range from 99.5% to 99.8% completeness and had a GC content of 66.5%.

**TABLE 1:** Genome features and the summary of assembling and annotation statistics

Parameter	C2	C4	C5	C8	C12
No. of raw reads (bp)	3,505,918	4,004,422	4,366,826	5,469,936	5,295,328
Total sequence length (bp)	4,670,871	4,635,204	4,447,264	4,639,930	4,441,390
Coverage (x)	40	46	54	64	68
Taxonomic classification					
ANI to the reference genome (%)	99.04	99.06	97.9	98.42	97.9
Genome assembly					
Total length (Mbp)	4.7	4.6	4.4	4.6	4.4
No. of scaffolds	130	240	179	207	181
*N*_50_ (kb)	71.2	48.7	46.8	44.1	50.6
*L*_50_	22	32	28	33	29
No. of total contigs	190	264	194	231	200
GC content (%)	66.5	66.5	66.5	66.5	66.5
BUSCO results					
Complete BUSCOs (%)	99.6	99.8	99.7	99.8	99.5
Single-copy and complete BUSCOs (%)	97.1	99.6	99.6	99.7	99.4
Duplicated and complete BUSCOs (%)	2.5	0.2	0.1	0.1	0.1
Fragmented BUSCOs (%)	0.2	0.1	0.3	0.1	0.3
Missing BUSCOs (%)	0.2	0.1	0.0	0.1	0.2
Genome annotation					
No. of predicted genes (total)	4,254	4,269	4,053	4,257	4,067
No. of coding DNA sequences (total)	4,180	4,195	3,981	4,184	3,995
No. of genes (coding)	4,164	4,171	3,948	4,156	3,967
No. of coding DNA sequences (with protein)	4,164	4,171	3,948	4,156	3,967
No. of RNAs	74	74	72	73	72
No. of rRNAs	4	4	4	4	4
No. of tRNAs	66	64	64	65	64

## Data Availability

This Whole Genome Shotgun project was deposited in GenBank under the accession number PRJNA993209. The versions described here are the first versions. The annotated genome sequences are available in GenBank via the following accession numbers: JAUKNN000000000 (C2), JAUKNO000000000 (C4), JAUKNP000000000 (C5), JAUKNQ000000000 (C8), and JAUKNR000000000 (C12). Raw reads were deposited in the NCBI Sequence Read Archive (SRA) under the accession numbers SRX21044479 (C2), SRX21044480 (C4), SRX21044481 (C5), SRX21044482 (C8), and SRX21044483 (C12).
